# Na-Faraday rotation filtering: The optimal point

**DOI:** 10.1038/srep06552

**Published:** 2014-10-09

**Authors:** Wilhelm Kiefer, Robert Löw, Jörg Wrachtrup, Ilja Gerhardt

**Affiliations:** 1 3. Physikalisches Institut, Universität Stuttgart, Stuttgart Research Center of Photonic Engineering (SCoPE), Pfaffenwaldring 57, D-70569 Stuttgart, Germany; 2 Center for Integrated Quantum Science and Technology (IQST), Pfaffenwaldring 57, D-70569 Stuttgart, Germany; 3 5. Physikalisches Institut, Universität Stuttgart, Pfaffenwaldring 57, D-70569 Stuttgart, Germany; 4 Max Planck Institute for Solid State Research, Heisenbergstraße 1, D-70569 Stuttgart, Germany

## Abstract

Narrow-band optical filtering is required in many spectroscopy applications to suppress unwanted background light. One example is quantum communication where the fidelity is often limited by the performance of the optical filters. This limitation can be circumvented by utilizing the GHz-wide features of a Doppler broadened atomic gas. The anomalous dispersion of atomic vapours enables spectral filtering. These, so-called, Faraday anomalous dispersion optical filters (FADOFs) can be by far better than any commercial filter in terms of bandwidth, transition edge and peak transmission. We present a theoretical and experimental study on the transmission properties of a sodium vapour based FADOF with the aim to find the best combination of optical rotation and intrinsic loss. The relevant parameters, such as magnetic field, temperature, the related optical depth, and polarization state are discussed. The non-trivial interplay of these quantities defines the net performance of the filter. We determine analytically the optimal working conditions, such as transmission and the signal to background ratio and validate the results experimentally. We find a single global optimum for one specific optical path length of the filter. This can now be applied to spectroscopy, guide star applications, or sensing.

Atomic vapours can be utilized for the construction of very narrow band-pass filters, and are commonly used in spectroscopy, astronomy and atmospheric sensing. Although described earlier^1^,^2^,^3^ for sun observations, these filters have been extensively explored in the 1990s^4^ and have been optimized since then^5^,^6^. Due to their atomic nature these filters will find naturally their way into atom based quantum optics applications. They are ideally suited to the extraction of narrow band and background free single photons matched to atomic transitions from single emitters, where the extraction of single photons or the detection of weak fluorescence signals often requires narrow-band filtering with high optical transmission^7^,^18^. A common alternative for this task is provided by Fabry-Pérot cavities. Unfortunately, these are sensitive to temperature drifts, and their overall transmission is limited by residual losses in the mirrors. Furthermore, their periodic transmission requires cascaded cavities to reject a wide range of background contributions.

Sodium is a particularly interesting atomic medium, since these filters allow for the research of the sodium layer of the atmosphere^8^,^9^,^15^. The sodium content in the mesosphere (approx. 90 km altitude) furthermore allows for the so-called sodium guide-stars, which can gain by an efficient filtering on the sodium wavelength. Only now, all solid-state lasers on the sodium-D-lines become available. Therefore, the field of efficient filtering on the sodium line will receive more interest in the near future. FADOFs based on atomic sodium were discussed already in the early 1990s^8^,^12^,^13^ and researched in detail later^10^,^14^. The ground state splitting of sodium amounts to 1.77 GHz. This implies that the Doppler spectrum (1.5 GHz for a single transition at 150°C) usually shows a single transition involving both ground states. Furthermore, the low vapour pressure introduces technical problems. These filters are generally heated to above 150°C, to reach the required vapour density, optical depth, and optical rotation. The magnetic field is required to be orders of magnitude higher than for atomic rubidium, or caesium. This is a result of the larger Doppler width and the smaller ground state splitting, such that a higher field is required to separate these states.

In this letter we describe a simulation and an experimental realization of a sodium-FADOF. The experimental implementation allowed for various magnetic fields and temperatures. Therefore, a full range of parameters can be researched, unlike in other schemes, where the required magnetic fields in the range of 200 mT are realized with permanent magnets. It is now possible to explore the optimal working conditions for the Na-FADOF. To date, these conditions are only derived empirically, and we are particularly interested to find these conditions in an analytical manner. Few papers evaluate the transmission properties against different parameters^18^,^19^,^20^. This multidimensional optimization problem requires a careful judgement of the specific disadvantages in optimizing a subset of parameters. The underlying math is discussed for the specific conditions with atomic sodium, but might be adapted to other atomic species, especially for the lighter alkalis (Li, Na, K), in which the ground states are spectrally overlapped with no magnetic field. A Li-FADOF is not reported to date, and it is questionable if it can be implemented due to the low vapour pressure. In particular the potassium FADOF requires high temperatures and magnetic fields, comparable to the sodium FADOF. Generally, the introduced optimization also applies to rubidium and caesium, but their ground state splitting (Rb: 3.5 GHz and 6.8 GHz, Cs: 9.1 GHz) is usually larger than their Doppler width.

## Methods

### Theoretical Background

**Figure 1:**
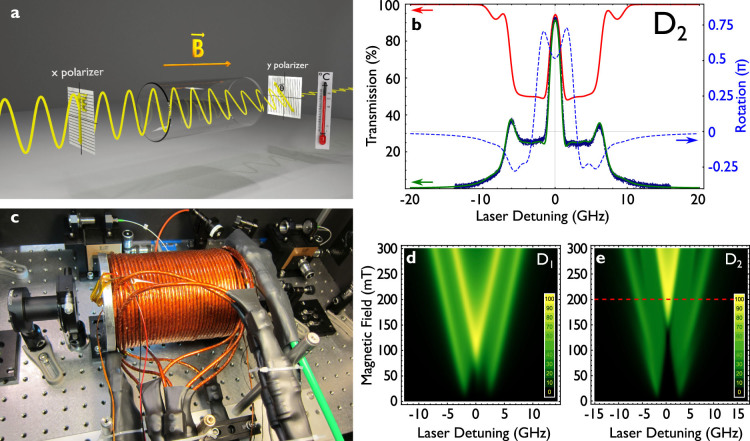
(a) general scheme of the discussed Faraday filter: an atomic vapour cell is placed between two crossed linear polarizers (wire-grid type shown). A magnetic field (B) leads to a Zeeman split of the involved levels, which then act differently on both circular polarization components. An overall turn of the polarization results in the transmission through the second polarizer (b) calculated (green) and measured (blue, dots) transmission spectrum of the D_2_ transition of sodium for 153°C and 0.2 T. Optical rotation (blue, dashed), Doppler spectrum (red, OD approx. 5). (c) experimental setup of Na-FADOF (d) density plot of the transmission for a temperature of 153°C for various magnetic fields, Na-D_1_transition. (e) same for the D_2_ transition. The red dashed line corresponds to the transmission spectrum in (b).

A FADOF consists of an atomic vapour cell, placed between two crossed linear polarizers (Fig. 1a). When an external magnetic field is applied along the optical axis, the Zeeman effect splits the atomic levels in an asymmetric manner due to a sign change in the Landé g-Factor between ground state and excited state. The σ^+^ and σ^−^ components of the linear polarized light, now experience different phase shifts when passing through the medium. This results in a net rotation of the linear polarization, such that the second, initially crossed, polarizer might transmit the light. Simultaneously, the absorption of light in the vapour cell has to be low. The ideal case is reached when the absorption is minimal and the rotation leads to a net 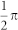 turn of the incident light.

For a simulation of the FADOF spectrum, the complex susceptibilities 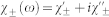 are calculated, which act on the two circular components. Since the spectra are calculated for a wide range of magnetic fields, the full Hamiltonian is expressed as *H* = *H*_0_ + *H_HFS_* +*H_Zeeman_*. It is required to diagonalize the full Hamiltonian, since the filter is neither operated only in the weak, nor in the strong magnetic field regime. Sodium has only one natural occurring isotope (^23^Na), therefore, 24, or 36 relevant susceptibilities for the Na-D_1_and Na-D_2_ line, respectively have to be taken into account. The situation is comparable to ^87^Rb, which exhibits the same spin structure. Since the atomic lines are temperature broadened (Doppler broadening, with a Voigt line profile), we calculate a full range of susceptibilities for each temperature. For simplicity, we do not assume different temperatures for the atomic density (which depends on the coldest point inside the sodium vapour cell) and the Doppler profile (which depends on the temperature of the atoms, which are under spectroscopy), as elsewhere^10^. A full derivation of the FADOF transmission is presented elsewhere^6^,^10^,^11^. In the case of sodium vapour in the absence of a magnetic field, the ground state splitting of Δ*v_g_* = 1.77 GHz leads to absorption spectra in which the two sub states are not resolved. At 153°C the Doppler width for each component already exceeds 2 GHz. Therefore, a FADOF around the sodium line centre requires a Zeeman splitting to separate the two sub-states.

Figure 1b shows a calculated transmission spectrum of the sodium FADOF for the D_2_-line (green). Zero detuning refers to the unperturbed sodium transition (3^2^*S*_1/2_ → 3^2^*P*_3/2_, centre of mass). Additional to this, the calculated Doppler transmission (red) and the relevant rotation (blue, dashed) are depicted. The Doppler spectrum shows two bands which are split from the band centre due to the Zeeman effect. These amount to an optical absorption of approx. 50% each, since each of them absorbs only one circular component. Note, that by passing through the cell the polarization is rotated and a modified absorption arises. The optical rotation can be represented by a projection of the Stokes vector in the Poincare sphere onto the y polarizer. Spectrally far from the band centre, the optical rotation amounts to zero and the light is effectively extinguished. The total FADOF transmission can be calculated as *T_tot_* = *T_Dopp_* · *T_rot_*, in which the transmission through the second polarizer can be calculated as 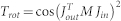 (Malus' law). Where we define the Jones vectors as 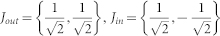. M is the relevant rotation matrix, defined as 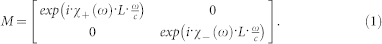
As an example: For a temperature of 153°C and with zero detuning, the optical transmission due to the Doppler absorption is close to unity (90.1%), and the optical rotation amounts to 0.52 π.

To evaluate the optimal working conditions of the FADOF operation, we calculated the full transmission spectra in a range of ±20 GHz for the sodium lines in respect to temperature, magnetic field, and length of the optical path. The corresponding source code (Mathematica 9.0, Wolfram Research) is supplied at http://gerhardt.ch/sodium.php, which was adapted from Ref. 6. Fig. 1d and 1e show the transmission of the filter as a function of the magnetic field at 153°C and a 100 mm long cell. The observed split of the ground states is equivalent to a Breit-Rabi diagram, and not fully symmetric. It is evident that the highest transmission for this temperature occurs close to the line centre, around zero detuning.

When calculating the optical transmission of the filter close to the line centre, one finds the dependency of the optical rotation *θ_F_* to the magnetic field, and the optical density. The optical susceptibilities 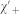 and 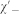 act on the circular components *σ*^+^ and *σ*^−^ respectively. The overall optical rotation can be described as: 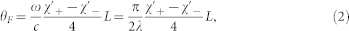
equivalent to the equation above (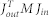).

Naively, one would think that simply by adapting the length of the filter *L*, and therefore changing *θ_F_* one could optimize the transmission in any case. Or, alternatively, by increasing the temperature, which increases the difference of the susceptibilities 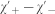. What is overlooked is the fact that the filter does not solely rely on the optical rotation, but also on the Doppler absorption in the atomic vapour cell. Therefore, if no sufficient magnetic field is supplied to separate the two components of the Doppler absorption, the filter only exhibits two lobes right and left of the Doppler broadened absorption line. Only when the field is larger transmission can occur between the two split transitions. Next, we distinguish between the two relevant components, which allow for the transmission of light through the filter: The rotation and the Doppler absorption of the atomic vapour.

Apart from the maximal transmission, another important quality measure for the filter is the equivalent noise bandwidth (ENBW), which describes the spectral pedestal vs. the peak intensity of the filter. This is equivalent to the inverse of the signal to noise ratio of the filter (
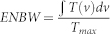
), when white background light is incident and we treat the point of maximum transmission as our signal, whereas the other part of the spectrum as a noise source. For narrowband signals in broadband noise, a filter achieves the signal-to-noise of an ideal filter with bandwidth ENBW^6^. Therefore, the ENBW of an optimal filter is as small as possible.

In summary it is required to achieve an optimum of three relevant parameters: a) the Doppler absorption in the cell has to be sufficiently small, b) the optical rotation should project a large amount to the y polarizer, and c) the side lobes of the peak transmission should be small, such that the ENBW is low.

### Experimental Implementation

To allow for an experimental verification of the above described theoretical approach, a Na-FADOF was set up. The experimental configuration is shown in Fig. 1c. For a magnetic field exceeding 400 mT with 250 A current, a solenoid was designed of polyimide (Kapton®) isolated hollow core copper tubing (2 × 4 mm). Seven layers of 35 windings each, with a void of 165 × 64 mm, allowed for the insertion of a vapour cell and a heater in various geometries. Cooling of the coil was realized by purging the layers alternating with approx. 17°C cold cooling water. 5 bar pressure resulted in a total throughput of 2 litres per minute. Only short-term experiments could be conducted at 250 A current, since from an operation current of larger than 235 A, the cooling water starts to boil after some minutes. The coil had a total resistance of 0.16 Ω. Two circular copper holders bring the vapour cell windows to the desired temperature and are themselves heated by two heat cartridges. The temperature was monitored inside the copper coils, on the outer rim of the quartz glass cell, and inside the copper blocks. The entire inset was thermally insulated by Teflon to the coil. The 100 mm long Na quartz glass cell (Suprasil) was produced in the university's own glass work shop. To compensate for residual losses in the cell windows, which were not AR-coated, we scaled the experimental data to match with the theoretical prediction. Initially, the experimental data had to be scaled by a factor of approximately 1.2, since about 85% of the off-resonant light could pass the cell. This was checked when a new cell was inserted into the experiment by measuring the incident laser power before and behind the cell. After some weeks of operation, the cells tend to darken, since sodium diffuses into the quartz windows and they have to be replaced. Then, the scaling factor was larger up to a factor of 3, which was an indication that the cells had to be replaced. This darkening can be circumvented, when specially coated cells are used^16^,^17^. As polarizers, a double Glan-Taylor polarizer was used at the input, and a polarizing beam-splitter at the exit port to allow for monitoring both polarization components (crossed-out and collinear with the first polarizer). By adding up the two output components, the Doppler transmission without rotation filtering was recorded.

As a light source, a dye ring laser, operated with Rhodamine 6G was utilized (Coherent 899-29, approx. 1 MHz line width). The beam had a diameter of 2.2 mm (1/e) and a Rayleigh range of approx. 3 m. The incident power was kept below 2 mW/cm^2^ (*I_sat_*~9.4 *mW*/*cm*^2^), and the typical fluctuations of the 30 GHz wide laser scan were renormalized to the laser power. The given maximal intensity accounts for a darkened cell. No effect of optical pumping was observed, as this would lead to asymmetries in the absorption spectra^24^,^25^. To characterize the transmission properties of the filter, such as temperature, magnetic field and polarisation, the incident light was stabilized to better than one percent peak-to-peak fluctuations at 1 s measurement time. These values show a very good match to all our simulations, which were performed for 150 Temperatures from 100–250°C, and a magnetic field variation from 0–300 mT. The level of agreement is maintained for the entire range of parameters depicted in the theoretical calculations.

For the magnetic field dependent measurement we scanned the magnetic field from 0 to 300 mT in 300 steps. This paper mainly describes the theoretical treatment, which is supported and verified by the experiments. The underlying math is derived by first principles and shows a very good agreement under other conditions^6^.

In the following we will rely for our discussion on the analytical results due to the excellent agreement of theory and experiment.

## Results and Discussion

To approach the optimal working conditions, namely the ratio of peak transmission with the lowest background contribution, it is preferable to have a transmission close to the line-centre. Otherwise the system cannot be used in conjunction with Na-resonant photons, and, as later outlined, the conditions are not preferable. We try to simultaneously optimize for two relevant parameters: peak transmission and low total transmitted background.

**Figure 2:**
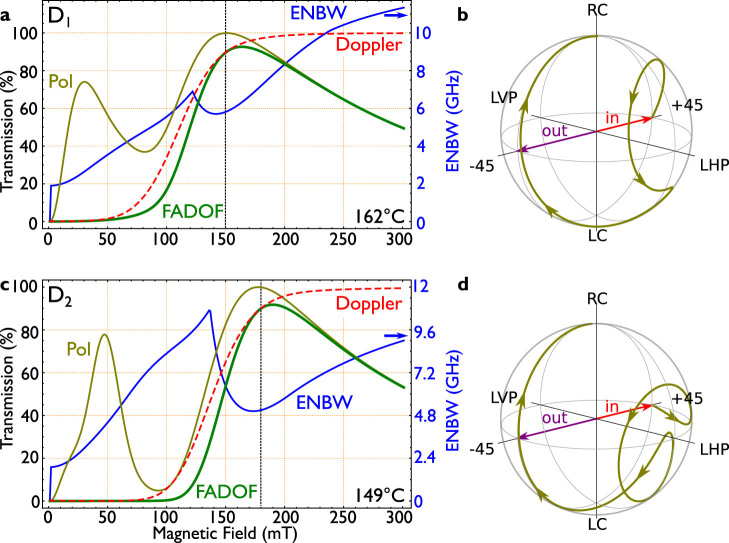
(a) Various contributions to the total FADOF transmission signal: Red, Dashed: Sodium D_1_-line, Doppler transmission for zero detuning, in resonance with the D_1_-line as a function of the magnetic fields. Effective transmission only due to the turned polarization (brown), and resulting FADOF transmission (green solid line) including Doppler transmission and rotation filtering. The equivalent noise bandwidth, describes the background contribution vs. the peak transmission (ENBW, blue). The optimal point for the given temperature is defined, where the ratio of the FADOF transmission and the ENBW is maximized (dashed, black vertical line). It lays between the minimum ENBW and the maximum for the FADOF transmission. (b) normalized representation of the optical rotation in the Poincare sphere with rising magnetic field. RC/LC: right and left-hand circular polarized light, LVP/LHP: linear vertical and horizontal polarized light. (c) and (d) same for the D_2_-line.

Fig. 2 displays the relevant components of the calculated FADOF spectrum for zero spectral detuning. These values are corresponding to a vertical line for zero detuning in Fig. 1d (D_1_) and Fig 1e (D_2_). With a rising magnetic field, both ground states split, and diverge from the centre (this can be best seen in Fig. 1d). Therefore, the Doppler transmission (red, dashed, *T_Dopp_*) rises with an increased magnetic field and an approximate sigmoidal behaviour is the consequence. The resulting absorption becomes negligible for all practical purposes, when the levels are sufficiently split, since the almost Gaussian absorption line results in an exponential confined Doppler signal. This holds, although the outlined theoretical approach proofs, that the resulting absorption line-shape is a mixture of the natural Lorentzian line and the Gaussian confined Doppler broadening. For the range of the optimal conditions, we do not observe a Lorentzian distribution in the transmission spectrum as observed elsewhere^26^. The optical dispersion, on the other hand, which leads to the optical rotation, scales as 1/*δ*^2^, i.e. much slower than the Doppler transmission under spectral detuning. This becomes evident, when one realizes that the susceptibilities can be approached by the derivative of the Lorentzian confined natural lines, which scales as 1/*δ*^2^. Therefore, it is possible to achieve a high optical rotation with still negligible Doppler absorption. For an increasing temperature, the Doppler width of a *transmission* line (0–100% transmission) changes proportionally to the temperature *T*, since both Doppler components widen as the temperature *T*. Please note, that this corresponds to a scaling behaviour proportional to 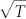 for an *absorption profile*, as the common Doppler broadening. Therefore, the position of the rising slope of the sigmoidal curve increases equivalently. The transition point changes accordingly proportional to the temperature *T*. Assuming the Doppler spectrum consists of two lines, which each absorb one circular component only, we can simulate the sigmoidal behaviour for zero detuning as follows: 
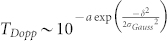
. Here, *a* represents a scale factor for the overall amount of absorption, which increases with the atomic density. *δ* is the spectral detuning (approximately rising linearly with the magnetic field), and *σ* the spectral line width of the Gaussian broadened line profile (which depends on the temperature).

Parallel to this, the polarization of the incident light is altered. This can be represented by a rotation of the Stokes vector in the Poincare sphere, as displayed in Fig. 2b and 2d. A comparable representation is presented for atomic Rubidium in Ref. 22. The initial polarizer and the analyser (by 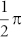 turned) are represented by two opposing Stokes vectors on the horizontal plane. Since both circular components can be treated independently, the resulting polarization state turns elliptical during propagation through the medium. Therefore, Fig. 2a and 2c show the projection of the polarization onto the analyser, which is the effective transmission (brown). Since the rotation results from the difference in the optical dispersion of both Zeeman components, we can approximate the rotation according to 
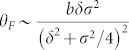
. This equation results from the assumption that the optical rotation acts on the two circular polarization components as the derivative of the Lorentzian line. Their difference corresponds to the optical rotation (see. Eqn. 2). Additional, the Zeeman effect splits the lines approximately linear. When it is assumed, that the lines are initially are at one position, it is possible to exchange the exact line position with a *δ*. In summary, *b* represents a scale factor for the density dependent rotation, *δ* the magnetic field dependent detuning from the centre of the spectrum, and *σ* the effective line width of the derivative of the (Lorentzian assumed) absorption line. The linear correspondence of the line position to the magnetic field in the high magnetic field regime was also found in Ref. 23. For atomic sodium the situation is even more predicted, due to the overlap of both ground states for zero magnetic field. As above, the overall FADOF-transmission can be calculated by multiplying the two individual transmission components. The total FADOF transmission is depicted in Fig. 2 (green). When the magnetic field is too low, the FADOF transmission is limited by the Doppler absorption, with rising magnetic field, the transmission increases and is reduced when the effective rotation deviates from 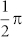, and increases further with a rising magnetic field.

The quality measure ENBW increases with a rising magnetic field, since the spectral pedestal increases. It further shows a discontinuity, and then exhibits another minimum (blue curve). From there, the ENBW continuously rises, since the overall spectrum broadens and therefore the ratio of the peak vs. the pedestal gets more and more disadvantageous. The discontinuity occurs when the peak is suddenly going over to another maximum, for example when the magnetic field rises. Unfortunately it is not straight forward to approximate the ENBW, such that the following equations describe the filter function.

To simultaneously optimize the transmission, while reducing the ENBW, we calculate the ratio of both components as a quality measure and maximize this value for all further considerations. This is equivalent to increasing the signal to noise ratio, when the aim is to detect light close to the sodium line center, on the peak transmission of the FADOF. The achieved maximum point (as shown by the dashed, vertical black line in Fig. 2) can be found between the minimum of the ENBW and the maximum for the transmission. This measure is considered as the optimal working conditions for the FADOF in all further calculations.

**Figure 3:**
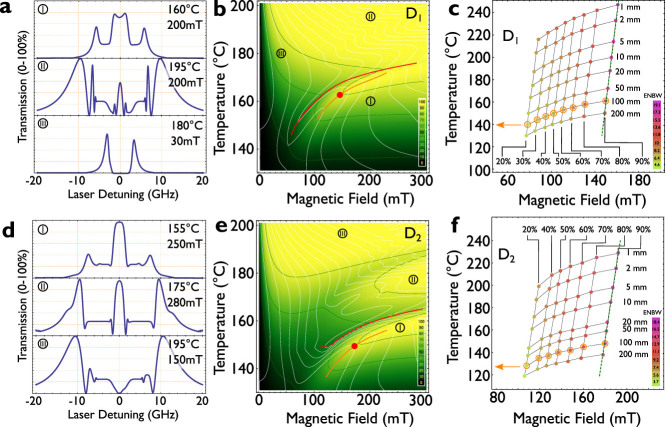
(a) different FADOF transmission spectra for different magnetic fields and temperatures. Labels correspond to the locations in b). (b) Density plot of the peak transmission of the D_1_-FADOF in dependence to the magnetic field, and the temperature (green lines denote 10, 50 and 90% transmission). Further lines indicate the ENBW (white, 1 GHz spacing), the optimal working conditions (orange), and the transition between a transmission peak at the line centre, respectively the outer rim of the Na-spectrum (red line). (c) summarized peak transmission and the ENBW in dependence of the length of the cell. The encircled points correspond the to the orange line in (b). (d) (e) and (f) same for the D_2_-line.

Since it is possible to calculate the filter and its optimal points for a variety of temperatures and magnetic fields, we plot the transmission and the ENBW for the entire parameter space, assuming a 10 cm long vapour cell. The calculation for the entire parameter range requires several days of computing time. In the online supporting material of this article two movies are supplied, which display the spectra for the entire parameter space, *B* vs. laser detuning *δ* for varying temperatures *T*. Fig. 3b (D_1_-line) and 3e (D_2_-line) depict the maximum filter transmission as a function of magnetic field and temperature in a colour scale. The maximum transmission per FADOF spectrum is depicted, independent of its exact spectral position. The resulting spectra are displayed in Fig. 3a and 3d. Green lines in Fig. 3b show the values for 10%, 50% and 90% transmission of the filter. This alone gives a certain minimal temperature to reach a minimum of 10% transmission: The D_1_-line FADOF has a minimum working temperature of 135°C, whereas the D_2_-line requires at least 120°C.

Further, the density plot is split into several domains: Two regions for the D_1_-line (top, and right bottom) and three regions for the D_2_-line (top left, right middle, and right bottom). This is because the FADOF exhibits several options to show a maximal transmission. For very low magnetic fields and high temperatures, when the levels are not split, the highest transmission occurs on the outer rim of the Doppler spectrum. Such a situation is depicted in Fig. 3aIII. This is not the desired working condition, since the filter should be resonant to the sodium transitions. At lower temperatures and a higher magnetic field, the filter exhibits one strong peak transmission close to the line centre (see e.g. Fig. 1b). The transition between the regions matches to the discontinuity of the ENBW plot, as shown in Fig. 2. This allows associating a maximum working temperature for the FADOF, when the transmission should be limited to the line centre (red line). The D_1_-line FADOF cannot be operated above 176.4°C, whereas the D_2_-line requires temperatures below 162°C.

The region below the red line in Fig. 3b and 3e represent the FADOF spectrum exhibits a transmission maximum close to the line centre. We now consider only the optimal conditions below the introduced upper limit. We apply the earlier measure for the optimal conditions. White lines depict the ENBW in 1 GHz steps. For each temperature there is an optimal point, which implies a high peak transmission and a minimal ENBW. All points form a joined curve, depicted in orange in Fig. 3b and 3e. These are the optimal points for each temperature and the corresponding magnetic field. Their spectral spread is below 100 MHz to the line centre of atomic sodium vapour. From a certain point on, a further increase of the temperature requires a higher magnetic field, which then leads to the situation, that the side peaks display the same transmission as the middle peak. This is equivalent to the maximum operating temperature as outlined before. When the points are plotted against the atomic vapour density, the line exhibits an approximately linear behaviour. This becomes clear, since the atomic vapour density scales roughly exponentially with the temperature. The spectral widening of the Doppler absorption is diminishingly small against the influence on the optical rotation. The curve for the optimal working conditions approaches to the maximum working temperature. The following equation is approximated to relate the temperature and the magnetic field. Since it is not possible to approximate the ENBW, unlike the transmission and rotation behaviour, we limit our findings to a numerically approached result only. It can be used to follow the optimal transmission conditions of the D_1_-line: 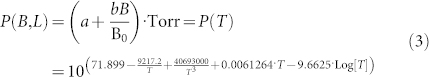

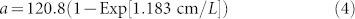

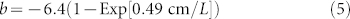

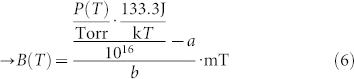


Here, *P*(*B*, *L*) = *P*(*T*) denotes the vapour density^10^,^21^ in Torr, *B(T)* the magnetic field in mT, *B*_0_ = 10^−3^ Tesla, *T* is the temperature in Kelvin, k is the Boltzmann constant and *L* is the length of the optical path (cell). The estimated equations for the optimal parameters of the D_2_-line are: 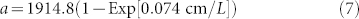

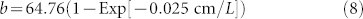


For the optimal points, we achieve an optical rotation around 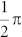 only. Higher order rotations usually lead to a deviation of the Stokes vector in the Poincare sphere from the fully crossed y polarizer, and therefore diminish the transmission. Also, this implies, that for another spectral detuning the rotation amounts to lower orders of n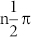, which results in more maxima right and left of the center peak.

One additional question is the optimal length of the vapour cell of the atomic filter. In Fig. 3c (D_1_-line, Fig. 3f for D_2_-line), the optimal conditions for the FADOF for different optical path length is shown in dependence to the temperature and magnetic field. The transmission is shown in percent (labelled) and the resulting ENBW is color-coded. The labelled line corresponds to the presented orange curve in Fig. 3b (D_1_) and 3e (D_2_). The shorter the cell is, the more it is required to heat up the cell to achieve a full 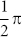 rotation. Therefore, the resulting Doppler spectrum is getting wider and the ENBW is increased. Subsequently, a longer cell is advantageous, but might be technically more challenging, since this requires a larger heater and homogeneous magnetic field. The relation between the length of the cell and the resulting temperature is approximately exponential, as the resulting vapour density in the cell. The exact position for one transmission value displays an approximate fixed ratio of magnetic field against temperature. This can be made clear by the change of the Doppler transmission (as in Fig. 2) when the temperature is increased. This changes proportionally with the temperature *T*, and can be approximated by a linear increase of the magnetic field with temperature with a factor of 8.1: 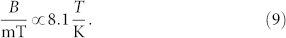
As before, the temperature *T* is in K and magnetic field *B* in mT. For the D_2_-line we get the following approximated connection: 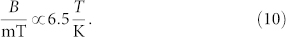
Both functions are marked by the green, dashed line in Fig. 3c and 3f.

**Figure 4:**
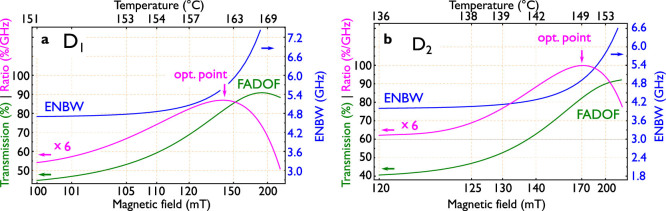
(a) ratio of FADOF transmission and ENBW, depicted for the optimum points from Fig. 3a. A global optimum point can be associated to the maximum of the quotient of the FADOF transmission and the ENBW. (b) same for the D_2_-line.

When the optimal points for each temperature and magnetic field combination are calculated, it is also possible to evaluate the resulting filter performance for each of the determined points. For all depicted optimal points for each temperature (orange, dashed line in Fig. 3b and 3e), Fig. 4 displays the total FADOF-transmission, the ENBW and their ratio, which was considered to give an indication of the optimal operating conditions as outlined above. By increasing the magnetic field and temperature it is possible to first increase the FADOF transmission, whereas the ENBW rises later, which is disadvantageous. The ratio shows one global maximum, which we consider as the optimal working conditions for the FADOF at the given optical path length (10 cm).

This point is for a 100 mm long sodium cell at 162°C and 144 mT for the D_1_-line FADOF. This point is labelled in Fig. 3b and 3e in red. For the D_2_-line, we estimate this point for 149°C and 170 mT.

## Conclusions

In this paper we have presented a complete analysis of a Na-FADOF and researched the system under various magnetic fields and temperatures. We find an excellent match between the theory and our experimental configuration. By a full diagonalization of the Hamiltonian, we are able to determine all relevant parameters for a wide temperature and magnetic field range. The calculated susceptibilities can be utilized to calculate a variety of filter lengths.

As a first indication a minimal and maximal temperature can be estimated to ensure a transmission above a certain fraction and to limit the transmission maximum to the spectral line centre. A fit function is given, which allows determining the optimal magnetic field for each cell temperature. When subsequently analysing the resulting curve, it becomes clear, that it is indeed possible to increase the peak transmission by increasing the magnetic field and temperature, but, from a certain point on, the pedestal of the transmission spectrum increases significantly. Therefore for a given experimental configuration, one global optimum can be associated.

The analysis of the cell length shows that a longer cell is generally preferable due to the lower Doppler width, and the lower ENBW.

As now powerful diode laser configurations are available for the sodium transitions, the sodium FADOF will allow to extract valuable spectral information and will allow for imaging in guide-star applications.

As common for FADOF filters, the supplied optical input field was linear polarized. Utilizing the introduced math, it will be possible to determine other orthogonal input and output states which allow for higher transmission and a better performance of the filter. We further envision establishing filter configurations with magnetic field gradients.

## Author Contributions

I.G. and W.K. envisioned and conducted the calculations and the experiment. R.L. helped in the planning and brought in important equipment. I.G. and J.W. supervised the team. All authors reviewed the manuscript.

## Supplementary Material

Supplementary Information Video Sodium D1 Line

Supplementary Information Video Sodium D2 Line
